# Insect-Active Toxins with Promiscuous Pharmacology from the African Theraphosid Spider *Monocentropus balfouri*

**DOI:** 10.3390/toxins9050155

**Published:** 2017-05-05

**Authors:** Jennifer J. Smith, Volker Herzig, Maria P. Ikonomopoulou, Sławomir Dziemborowicz, Frank Bosmans, Graham M. Nicholson, Glenn F. King

**Affiliations:** 1 Institute for Molecular Bioscience, The University of Queensland, Brisbane, QLD 4072, Australia; jennifer.smith@imb.uq.edu.au (J.J.S.); v.herzig@imb.uq.edu.au (V.H.); maria.Ikonomopoulou@qimrberghofer.edu.au (M.P.I.); 2 School of Life Sciences, University of Technology Sydney, NSW, Sydney 2007, Australia; slawomirdziemborowicz@gmail.com (S.D.); graham.nicholson@uts.edu.au (G.M.N).; 3 Department of Physiology & Solomon H. Snyder Department of Neuroscience, Johns Hopkins University, School of Medicine, Baltimore, MD 21205, USA; frankbosmans@jhmi.edu

**Keywords:** insecticide, pharmacology, venom, sodium channel, calcium channel, spider

## Abstract

Many chemical insecticides are becoming less efficacious due to rising resistance in pest species, which has created much interest in the development of new, eco-friendly bioinsecticides. Since insects are the primary prey of most spiders, their venoms are a rich source of insect-active peptides that can be used as leads for new bioinsecticides or as tools to study molecular receptors that are insecticidal targets. In the present study, we isolated two insecticidal peptides, µ/ω-TRTX-Mb1a and -Mb1b, from venom of the African tarantula *Monocentropus balfouri.* Recombinant µ/ω-TRTX-Mb1a and -Mb1b paralyzed both *Lucilia cuprina* (Australian sheep blowfly) and *Musca domestica* (housefly), but neither peptide affected larvae of *Helicoverpa armigera* (cotton bollworms). Both peptides inhibited currents mediated by voltage-gated sodium (Na_V_) and calcium channels in *Periplaneta americana* (American cockroach) dorsal unpaired median neurons, and they also inhibited the cloned *Blattella germanica* (German cockroach) Na_V_ channel (BgNa_V_1). An additional effect seen only with Mb1a on BgNa_V_1 was a delay in fast inactivation. Comparison of the Na_V_ channel sequences of the tested insect species revealed that variations in the S1–S2 loops in the voltage sensor domains might underlie the differences in activity between different phyla.

## 1. Introduction

Spider venoms contain a plethora of bioactive peptides that target a diverse range of vertebrate and invertebrate voltage-gated ion channels [1,2]. Spider venoms evolved for two main purposes: defense against predators [3] and as a chemical weapon for prey capture [4,5]. Since insects are the predominant prey of most spiders, these arachnids have developed a range of toxins that are highly effective at incapacitating insects [6]. There are over 46,600 extant spider species [7], with the venoms of some spiders containing >1000 different peptides [8]. Thus, spider venoms are an ideal source of toxins that can be used to study insect ion channels, or as potential candidates for the development of insecticides [6,9]. The two major pharmacological targets of spider-venom peptides are voltage-gated calcium (Ca_V_) channels and voltage-gated sodium (Na_V_) channels [6,10]. Both channels consist of four homologous domains (DI–DIV), each of which contains six transmembrane helices (S1–S6). The four sets of S5–S6 helices come together to form the pore of the channel, while each S1–S4 region serves as an independent voltage sensor [11,12]. Many chemical insecticides target insect Na_V_ channels and bind to the S5–S6 pore region, but these insecticides are becoming less useful due to increasing resistance in pest species [13,14,15,16]. However, spider venoms represent a source of novel toxins that act on insect Na_V_ channels via a different mode of action to extant chemical insecticides. The majority of spider toxins are gating modifiers that interact with the voltage-sensing domains and not the pore region, and therefore pests resistant to current Na_V_ channel insecticides would likely be sensitive to spider toxins [17]. In addition to Na_V_ channels, insect Ca_V_ channels are an emergent insecticidal target, and they are another major target of spider toxins [9,18]. Indeed, a spider toxin that inhibits insect Ca_V_ channels has been developed into a bioinsecticide called SPEAR^TM^ that will become commercially available early 2017 [19].

This study describes the isolation, recombinant production, and characterization of the disulfide-rich peptides µ/ω-TRTX-Mb1a and µ/ω-TRTX-Mb1b (hereafter Mb1a and Mb1b) from venom of the African tarantula *Monocentropus balfouri*. Both peptides inhibited Na_V_ and Ca_V_ channel currents in cockroach neurons as well as the cloned Na_V_ channel from the German cockroach *Blatella germanica*. Mb1a and Mb1b are paralytic to the dipterans *L. cuprina* and *M. domestica*, but do not affect the lepidopteran *H. armigera*. We propose that variations between taxa in the S1–S2 loops of the Na_V_ channel voltage-sensor domains underlie the differences in activity between phyla and mode of action of Mb1a and Mb1b. Ultimately, understanding how spider toxins bind to insect Na_V_ and Ca_V_ channels will facilitate the rational design of bioinsecticides with better toxicity and selectivity profiles than the current arsenal of chemical insecticides.

## 2. Results

### 2.1. Isolation and Sequencing of µ/ω-TRTX-Mb1a and -Mb1b

**Figure 1:**
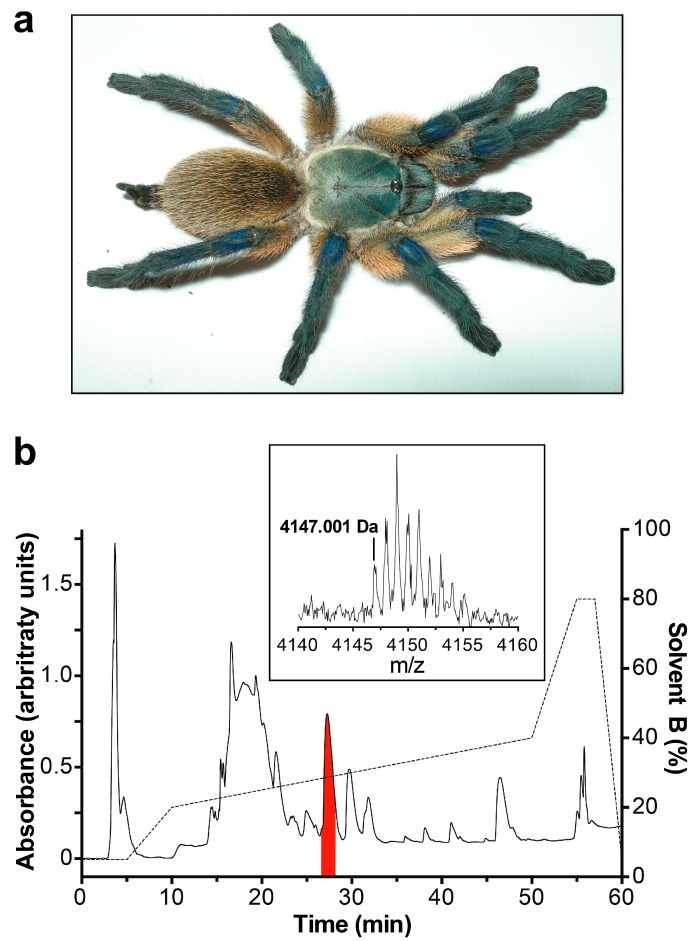
(**a**) Photo of a female *M. balfouri*; (**b**) Chromatogram resulting from RP-HPLC fractionation of *M. balfouri* venom. The peak highlighted in red contains the µ/ω-TRTX-Mb1a/b peptide. The dotted line indicates the gradient of solvent B (90% acetonitrile/0.1% formic acid). Inset is a MALDI-TOF mass spectrum of the isolated µ/ω-TRTX-Mb1a/b peptide.

A screen of insecticidal activity by injection into sheep blowflies of fractions resulting from reversed-phase (RP) HPLC separation of *M. balfouri* venom revealed that the fraction eluting at ~35% solvent B concentration (Figure 1) induced paralysis that was reversible within 24 h. Mass spectrometry revealed that this fraction consists of a single peptide with monoisotopic (M + H^+^) mass 4147.00 Da (Figure 1, inset). N-terminal sequencing of this peptide revealed the first 36 residues as GVDKPGCRYMFGGCVQDDDCCPHLGCKRKGLYCAWD(A)(T), with residues 37 and 38 shown in parentheses due to sequencing ambiguities. The two C-terminal residues cannot be both A and T as this would yield a sequence whose mass does not correspond to that of the observed peptide. Therefore, two peptide sequences were derived that matched the observed mass, one with ′GT-NH_2_′ as the C-terminus and another with ′AS-NH_2_′. In an attempt to ascertain the two terminal residues, LC-MS/MS was performed on a nine-residue C-terminal fragment of reduced and alkylated native peptide liberated by tryptic digestion. Analysis of the b- and y-ion series revealed three matches for ′GT-NH_2_′ while only one match corresponded to ′AS-NH_2_′ therefore ′GT-NH_2_′ is the most likely C-terminal sequence of the native peptide. Nevertheless, both peptide sequences were expressed for further characterization as the native sequence was not conclusively determined. Based on their activity (see below) and the species of origin, the two peptides were named in accordance with published nomenclature guidelines [20] as µ/ω-TRTX-Mb1a (sequence GVDKPGCRYMFGGCVQDDDCCPHLGCKRKGLYCAWDGT) and µ/ω-TRTX-Mb1b (sequence GVDKPGCRYMFGGCVQDDDCCPHLGCKRKGLYCAWDAS).

### 2.2. Recombinant Production of Mb1a and Mb1b

**Figure 2:**
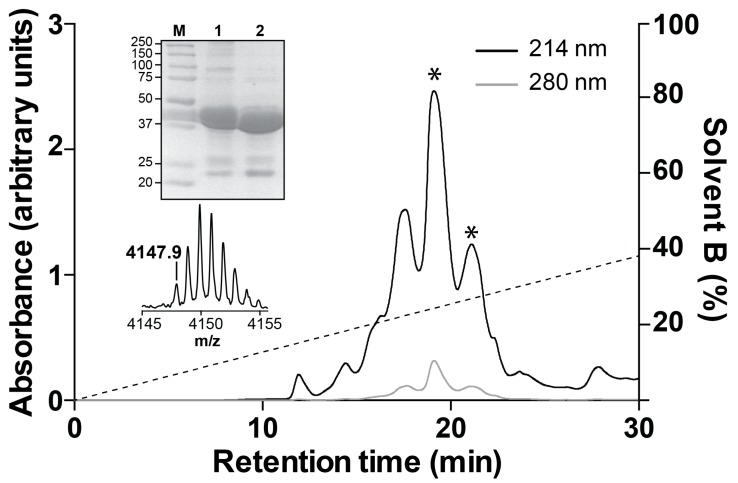
Recombinant production of Mb1a. Semi-preparative RP-HPLC chromatogram of recombinant Mb1a released by TEV protease cleavage of the MBP-Mb1a fusion protein (see Materials and Methods for more details). The dotted line indicates the gradient of solvent B (90% acetonitrile/0.043% TFA). Top inset: SDS-PAGE gel showing pre-cleaved MBP-Mb1a fusion protein (lane 1) and remaining MBP after cleavage (lane 2). Lane M contains molecular markers (masses in kDa). Bottom inset: MALDI-TOF mass spectrum of pure recombinant Mb1a.

Recombinant Mb1a and Mb1b were produced by overexpression in the periplasm of *Escherichia coli* using the system we previously optimized for expression of disulfide-rich venom peptides [21]. The fusion protein was the major soluble protein expressed after induction, and cleavage of the fusion protein with tobacco etch virus (TEV) protease liberated free recombinant Mb1a (Figure 2, top inset) and Mb1b (not shown). RP-HPLC purification of the cleaved peptides resulted in elution profiles consisting of two peaks (Figure 2) with the correct mass (*m*/*z* calculated: 4147.8 Da, observed: 4147.9 Da) (Figure 2, bottom inset). Re-injection of each peak separately resulted in the reappearance of two peaks (data not shown), suggesting possible *cis*-*trans* isomerization of the proline residue at position 5 or 22. The final yield for both peptides was ~100 μg per litre of bacterial culture. The recombinant peptides were used for all in vitro and in vivo assays.

### 2.3. Insecticidal Activity of µ/ω-TRTX-Mb1a and -Mb1b

**Figure 3:**
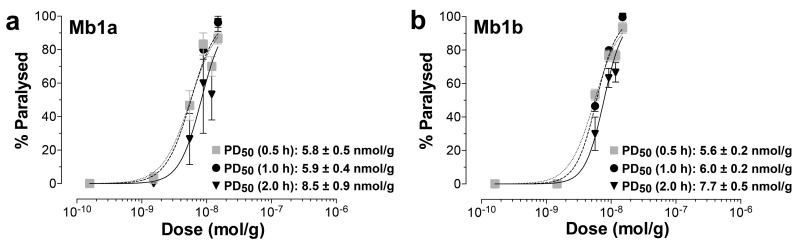
Insecticidal effects of recombinant (**a**) Mb1a and (**b**) Mb1b. Toxins were injected intra-thoracically into *L. cuprina* blowflies and paralytic effects measured at 0.5, 1, 2 and 24 h post injection. PD_50_ values for each time-intervals (±S.E.M.) are indicated. Hill slopes for Mb1a were 2.01 (0.5 h), 2.24 (1.0 h), 2.53 (2.0 h), and for Mb1b: 2.23 (0.5 h), 2.74 (1.0 h), 2.98 (2.0 h). No lethality was observed, and the paralytic effects caused by both toxins were fully reversible within 24 h.

Both Mb1a and Mb1b caused fast, but fully reversible, paralysis of sheep blowflies (*L. cuprina*) with median paralytic dose (PD_50_) values in the range of 5600–5800 pmoL/g at 30 min post-injection (Figure 3). No lethal effects were observed, and paralysis was fully reversed after 24 h. Complete paralysis was also seen for both Mb1a and Mb1b at 60 min post-injection into house flies (*M. domestica*), with partial recovery observed after 24 h (Mb1a: 3/5 recovered; Mb1a: 4/5 recovered). In contrast, injection of up to 73.4 nmoL/g of Mb1b into cotton bollworms (*H. armigera* larvae) did not induce any paralytic effects. No lethality or significant changes in the weight gain of the larvae occurred within the 72-h observation period.

### 2.4. Activity of Mb1a and Mb1b on Insect Na_V_ Channel Currents

**Figure 4:**
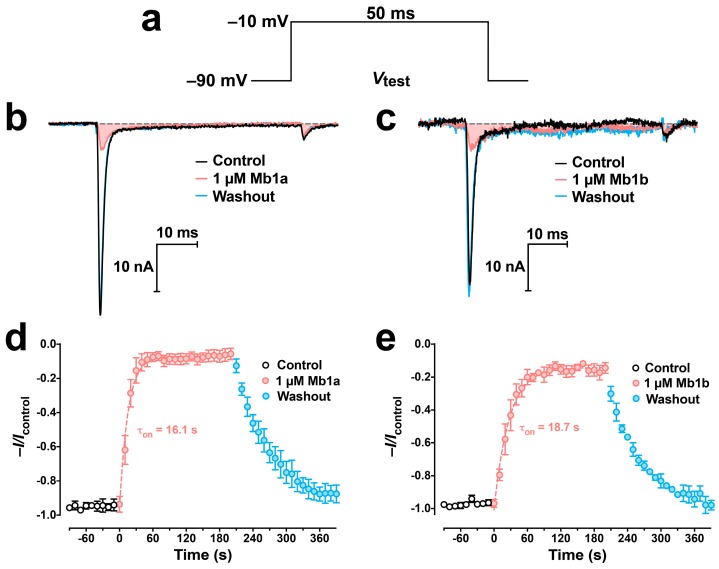
Effects of 1 μM Mb1a and Mb1b on *P. americana* DUM neuron *I*_Na_. (**a**) The depolarizing voltage test protocol (*V*_test_) used to elicit *I*_Na_ at 0.1 Hz. (**b**,**c**) Representative superimposed current traces elicited by *V*_test_ prior to (black lines), and 3 min following, exposure to 1 μM Mb1a ((**b)**, red line) and Mb1b (**c**, red line). Solid gray lines represent *I*_Na_ recorded 3 min after perfusion with toxin-free solution, while dashed gray lines represent zero current. (**d**,**e**) Timecourse of the block of *I*_Na_ by 1 µM Mb1a (**d**) and Mb1b (**e**). Average normalized peak *I*_Na_ before (open circles), during (red circles), and after (blue circles) perfusion with 1 μM toxin. Values represent the mean ± S.E.M of 5 (**d**) or 3 (**e**) experiments. The rate constant for association of the toxin to the channel (τ_on_) was calculated using Equation (1) defined in the Materials and Methods. There was no significant difference in current inhibition by Mb1a and Mb1b (*p* = 0.169).

At a concentration of 1 μM, both Mb1a (Figure 4a) and Mb1b (Figure 4b) reversibly inhibited endogenous sodium channel currents (*I*_Na_) in dorsal unpaired median (DUM) neurons isolated from the American cockroach *Periplaneta americana*. Mb1a rapidly inhibited 91.5 ± 3.0% of the current (*n* = 5; τ_on_ = 16.1 s; Figure 4b,d), while Mb1b was slightly less potent, inhibiting current by 84.3 ± 3.7% (*n* = 3; τ_on_ = 18.7 s; Figure 4c,e). Tail currents were unaffected by Mb1a and Mb1b (Figure 4b,c).

**Figure 5:**
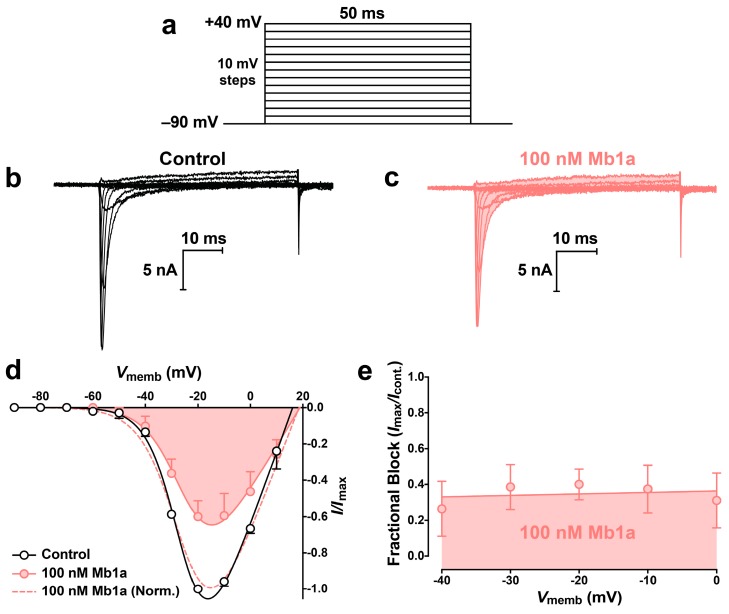
Effect of 100 nM Mb1a on the voltage-dependence of activation of *P. americana* DUM neuron Na_V_ channels. (**a**) Families of Na_V_ channel currents were elicited by depolarizing test pulses to +40 mV from a holding potential of −90 mV in 10-mV steps. Representative superimposed families of *I*_Na_ are shown prior to (**b**), and 5 min after (**c**), application of 100 nM Mb1a. Currents were generated using the test pulse protocol shown in panel a. (**d**) Normalised *I*_Na_-*V* relationships before (open circles), and after (red circles and shaded), application of 100 nM Mb1a. Data were fitted using Equation (1) (see Materials and Methods). Currents recorded in the presence of toxin were normalised against maximum peak *I*_Na_ in controls (red solid curve) and maximum peak *I*_Na_ in the presence of toxin (red dashed curve). (**e**) Linear regression analysis of the data using Equation (2) (see Materials and Methods) revealed that inhibition of *I*_Na_ was voltage-independent over the range −40 to 0 mV in the presence of 100 nM Mb1a. Data points are the mean ± S.E.M of 3 experiments.

To investigate if current inhibition was caused by a toxin-induced shift in the voltage- dependence of Na_V_ channel activation, *I*_Na_/*V* curves were generated before (Figure 5b) and after (Figure 5c) addition of 100 nM Mb1a. No shift in *I*_Na_/*V* curves was observed following perfusion with toxin (Figure 5d). To determine if toxin-induced block was voltage-dependent, the peak current in the presence of 100 nM Mb1a was plotted as a percentage of the control current (Figure 5e). There was no significant change in the extent of inhibition for *I*_Na_ depolarizations ranging from −40 mV to 0 mV, indicating that toxin-induced block is voltage-independent over this range.

**Figure 6:**
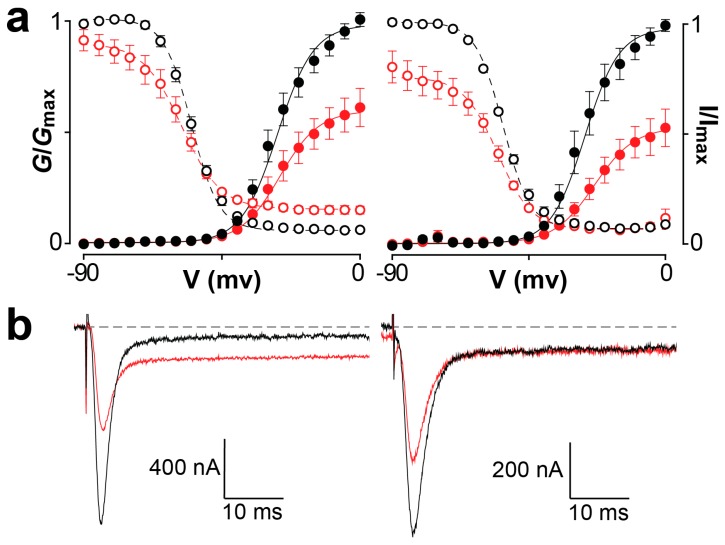
(**a**) Effect of 200 nM Mb1a (left) or Mb1b (right) on BgNa_V_1. Normalized conductance- voltage (G-V) relationships (G/G_max_) are shown by closed circles and steady-state inactivation (SSI) relationships (*I*/*I*_max_) by open circles, before (black) and after (red) toxin addition. Normalization was performed relative to the peak current before toxin addition. Solid and dashed lines depict, respectively, the G-V and SSI curves fit using the standard Boltzmann equation. Oocytes were depolarized in 5-mV steps from a holding potential of –90 mV up to 5 mV for 50 ms, followed by a depolarizing pulse to −15 mV for 50 ms. Peak current from the initial step series was converted to conductance and normalized to create the G-V relationship while peak current from the following –15 mV voltage depolarization step was normalized to yield the SSI relationship. Mb1a caused a decrease in peak current and increase in persistent current, while Mb1b reduced peak current without affecting persistent current (*n* = 4; error bars represent S.E.M) (**b**) Representative examples of the effects of Mb1a (left) and Mb1b (right) on BgNa_V_1 current when depolarized to –15 mV, with black and red traces corresponding to the current before and after toxin application, respectively. Each set of traces is taken from an individual oocyte used to generate the data shown in panel (**a**). Note that the persistent current seen in the traces in panel (**b**) does not result from inhibition of fast inactivation by Mb1a and Mb1b since BgNav1 inherently possesses these characteristics at mildly depolarizing voltages [22,23,24].

Mb1a and Mb1b were also tested on the cloned BgNa_V_1 channel from the German cockroach (*Blattella germanica*), heterologously expressed in *Xenopus* oocytes. (Note that the orthologous *P. americana* Na_V_ channel has never been functionally expressed in oocytes.) Similar to their activity on American cockroach Na_V_ channels, application of Mb1a or Mb1b to BgNa_V_1 caused a reduction in peak BgNa_V_1 currents with no shift in the G_Na_-V curve (Figure 6a). However, application of Mb1a caused inhibition of fast inactivation, which was not seen with Mb1b (Figure 6b). Therefore, the C-terminal residues ′GT′ are responsible for the inhibition of fast inactivation seen with Mb1a, likely mediated by an additional interaction with the domain IV voltage-sensor [12].

### 2.5. Effect of Mb1a and Mb1b on Insect Ca_V_ Channel Currents

**Figure 7:**
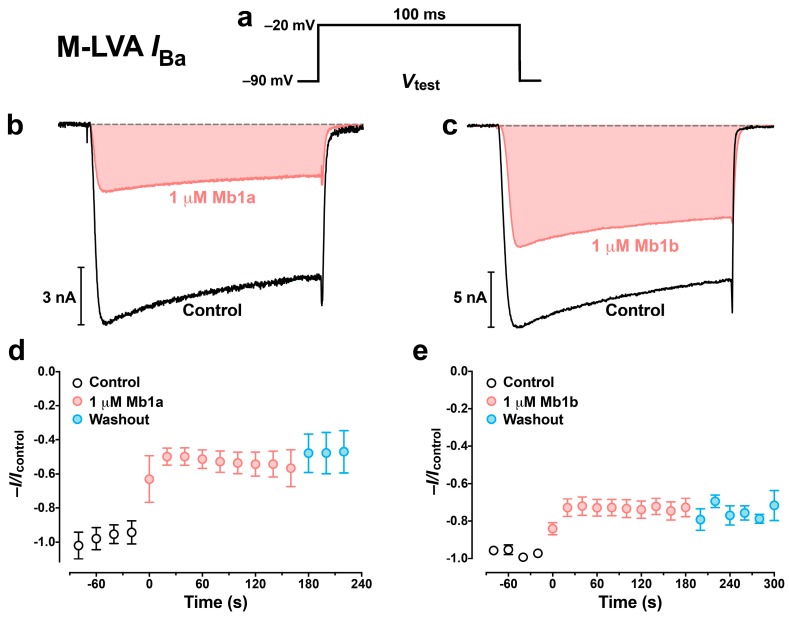
The effects of 1 μM Mb1a and Mb1b on *P. americana* M-LVA Ca_V_ channel currents. (**a**) M-LVA *I*_Ba_ were elicited by 100-ms depolarizing test pulses to –20 mV from a holding potential of –90 mV. (**b**,**c**) Representative superimposed M-LVA *I*_Ba_ showing tonic block of M-LVA Ca_V_ channel currents before (black lines) and following (red lines) a 3 min perfusion, with (**b**) 1 µM Mb1a and (**c**) 1 µM Mb1b. Dashed gray lines represent zero current. (**d**,**e**) Timecourse of inhibition of normalized peak M-LVA Ca_V_ channel currents by (**d**) 1 µM Mb1a and (**e**) 1 µM Mb1b. Data are mean ± S.E.M, *n* = 4. Average normalized peak *I*_Ba_ before (open circles), during (red circles), and after (blue circles) perfusion with 1 μM toxin. Data are mean ± S.E.M of 5 (panel **d**) or 3 (panel **e**) experiments. Inhibition of *I*_Ba_ by Mb1a was significantly greater than for Mb1b (*p* = 0.008).

**Figure 8:**
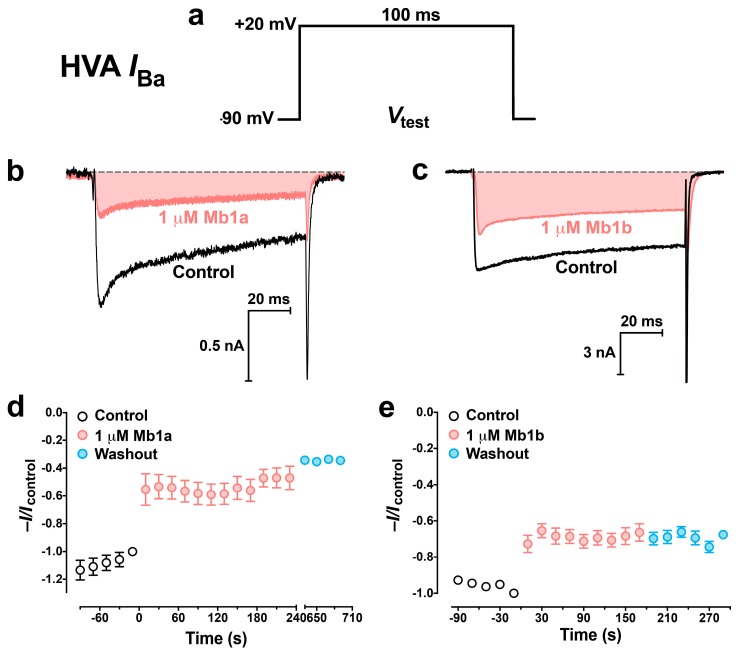
Effect of 1 μM Mb1a and Mb1b on *P. americana* HVA Ca_V_ channel currents. (**a**) M-LVA *I*_Ba_ were elicited by 100-ms depolarizing test pulses to +20 mV from a holding potential of −90 mV. (**b**,**c**) Representative superimposed HVA *I*_Ba_ showing tonic block of HVA Ca_V_ channel currents before (black lines) and following a 3 min perfusion (red lines) with (**b**) 1 µM Mb1a and (**c**) 1 µM Mb1b. Dashed gray lines represent zero current. (**d**,**e**) Timecourse of inhibition of normalized peak HVA Ca_V_ channel currents by (**d**) 1 µM Mb1a and (**e**) 1 µM Mb1b. Data are mean ± S.E.M, *n* = 4. Average normalized peak *I*_Ba_ before (open circles), during (red circles), and after (blue circles) perfusion with 1 μM toxin. Data are mean ± S.E.M of 5 experiments (panel **d**) or 3 experiments (panel **e**). Inhibition of *I*_Ba_ by Mb1a was significantly greater than for Mb1b (*p* = 0.007).

**Figure 9:**
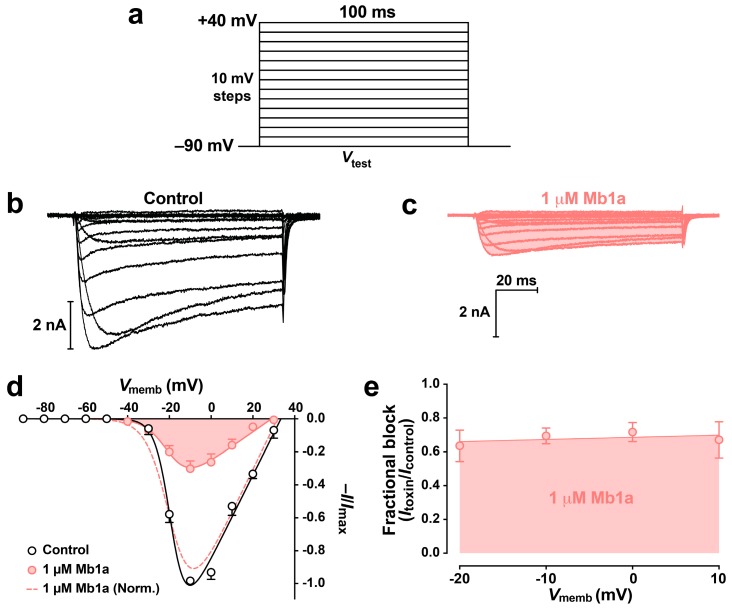
Effect of 1 µM Mb1a on the voltage-dependence of activation of *P. americana* DUM neuron Ca_V_ channels. (**a**) Families of Ca_V_ channel currents were elicited by depolarizing test pulses to +40 mV from a holding potential of –90 mV in 10-mV steps. Representative superimposed families of *I*_Na_ are shown prior to (**b**), and 5 min after (**c**), application of 1 µM Mb1a. Currents were generated using the test pulse protocol shown in panel **a**. (**d**) Normalised *I*_Ba_-*V* relationships before (open circles) and after (red circles and shaded) application of 1 µM Mb1a generated using the pulse protocol in panel **a**. Data were fitted using Equation (2) (see Materials and Methods). Currents recorded in the presence of toxin were normalised against maximum peak *I*_Ba_ in controls (red solid curve) and maximum peak *I*_Ba_ in toxin (red dashed curve). (**e**) Linear regression analysis of the data using Equation (2) (see Materials and Methods) revealed that inhibition of *I*_Ba_ by 100 nM Mb1a was voltage-independent over the range –40 to +10 mV. Data points are mean ± S.E.M, *n* = 4.

At a concentration of 1 μM, Mb1a and Mb1b rapidly and irreversibly inhibited both mid-/low-voltage-activated (M-LVA) and high-voltage-activated (HVA) Ca_V_ channel currents endogenously present in *P. americana* DUM neurons (Figure 7 and Figure 8). Mb1a was slightly more potent than Mb1b on both types of Ca_V_ channel currents, with 45% of M-LVA Ca_V_ channel currents and 48% of HVA Ca_V_ channel currents inhibited by Mb1a (Figure 7b and Figure 8b, respectively) compared with 27% inhibition of M-LVA currents and 34% inhibition of HVA Ca_V_ channel currents by Mb1b (Figure 7c and Figure 8c, respectively). Mb1a did not shift the *I*_Ba_/*V* curve (Figure 9a–d), and its inhibition of Ca_V_ channel currents was voltage-independent (Figure 9e).

## 3. Discussion

### 3.1. Promiscuous Pharmacology of Mb1a/1b

**Figure 10:**
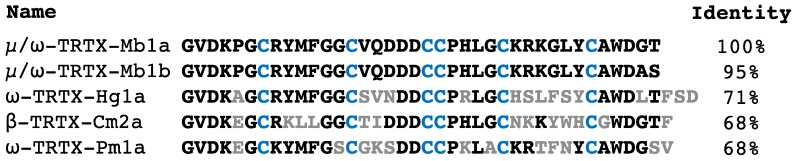
Sequence alignment of Mb1a and Mb1b with their closest homologues. Amino acids identical to Mb1a are in bold and differences are highlighted in gray. Cysteines are coloured blue.

The closest homologues of Mb1a and-Mb1b are the spider toxins ω-TRTX-Hg1a (71% identity) [25,26], β-TRTX-Cm2a (68% identity) [27] and ω-TRTX-Pm1a (68% identity) [28] (Figure 10), all of which are active on vertebrate voltage-gated ion channels. None of these toxins have been tested against insects. It is interesting to note that the Ca_V_ channel inhibitors ω-TRTX-Hg1a and ω-TRTX-Pm1a also modulate Na_V_ channel currents by reducing peak current and delaying fast inactivation [28,29], akin to the action of Mb1a on BgNa_V_1 (Figure 6).

The C-terminus of ω-TRTX-Pm1a was found to be critical for its activity: a C-terminally truncated analogue was almost inactive on both Ca_V_ and Na_V_ channels [28]. Similarly, we found that the activity of Mb1a/1b was influenced by the C-terminal region, with the putative native toxin, Mb1a, being more potent at inhibiting Ca_V_ channels than Mb1b. Furthermore, the two C-terminal residues of Mb1a are responsible for its inhibition of fast inactivation in BgNa_V_1, as this was not observed with Mb1b. It is interesting that the delay in inactivation appears to be simply due to the loss and/or addition of a methyl group of the side chains of G and T, respectively, compared to those of AS. The ability of Mb1a to *inhibit* peak BgNa_V_1 currents and delay fast inactivation contrasts with that of the toxin PnTx4(5-5) from *Phoneutria nigriventer*, which *enhances* peak current in addition to causing delayed inactivation of BgNa_V_1 [30].

**Figure 11:**
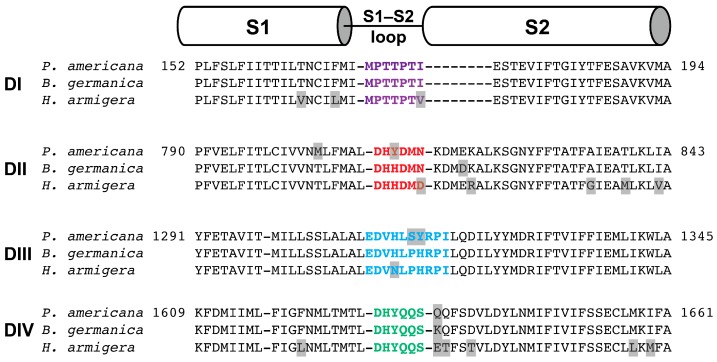
Alignment of the S1–S2 regions from each of the four domains (DI–DIV) in the Na_V_ channels from *P. americana* (UniProt D0E0C1), *B. germanica* (UniProt O01307), and *H. armigera* (deduced from the published genome). The S1–S2 extracellular loops from the four domains are coloured purple (DI), red (DII), blue (DIII) and green (DIV). Differences in amino acid sequence between species are highlighted in grey. The boundaries of S1 and S2 are based on the recently determined structure of the *P. americana* Na_V_PaS channel [39].

Fast inactivation of Na_V_ channels is mediated by domain IV, and spider toxins interacting with the S3–S4 extracellular loop in channel domain IV (DIV) have been found to inhibit fast inactivation [31,32]. The loss of inhibition of fast inactivation by Mb1b suggests that the C-terminal ′GT′ residues in Mb1a facilitate its interaction with the domain IV voltage sensor of BgNa_V_1. Notably, Mb1a did not delay fast inactivation of *P. americana* Na_V_ channel currents, even though the DIV S3–S4 DIV loop is identical in the *P*. *americana* and *B. germanica* Na_V_ channels. However, numerous recent studies have revealed that the extracellular S1–S2 loops can be important sites for toxin recognition [24,33,34,35,36,37,38]. A comparison of the DIV S1–S2 region of the *P. americana* and *B. germanica* channels reveals only a single difference (K1634Q; Figure 11), which may be at least partly responsible for the ability of Mb1a to inhibit the fast inactivation of *B. germanica* Na_V_ channels.

Inhibition of peak *I*_Na_ without a shift in the *I*_Na_/*V* curve, as seen with Mb1a and Mb1b, may indicate a simple pore blocking mechanism of action. However, most Na_V_ channel spider toxins are gating modifiers [11,40] that interact with the voltage sensor domains [11], and numerous spider toxins such as μ-TRTX-Hs2a (huwentoxin IV; [41]) and μ-TRTX-Hd1a [42] have been found to inhibit Na_V_ channels by interacting with the DII voltage sensor without causing a shift in the voltage-dependence of channel activation. Therefore, it is likely that Mb1a and Mb1b inhibit insect Na_V_ channels via gating modification. Compared to Na_V_ channels, much less is known about how spider toxins interact with Ca_V_ channels [43]. Spider toxins that affect Ca_V_ channels are generally thought to act as gating modifiers, although some toxins such as ω-AGTX-Aa3a and ω-SGTX-Sf1a are believed to be pore blockers [44,45,46]. Mb1a and Mb1b both inhibit peak Ca_V_ channel currents without shifting the *I*_Ba_/*V* curve, consistent with them being pore blockers. However, further experiments will be required to determine the binding sites of these two peptides on insect Ca_V_ and Na_V_ channels.

### 3.2. Phyletic Selectivity of Mb1a/1b

Several spider toxins have been found to paralyze or kill the dipterans *L. cuprina* and *M. domestica*, with activities ranging from 198 pmoL/g (LD_50_; U_1_-AGTX-Ta1a) to 2229 pmoL/g (PD_50_; μ-SGTX-Sf1a) on *L. cuprina*, and 77 pmoL/g (LD_50_; ω-HXTX-Hv1a) to 1380 pmoL/g (LD_50_; μ-AGTX-Aa1b) on *M. domestica* [47,48,49,50]. With PD_50_ values of 5600–5800 pmoL/g, Mb1a and Mb1b are only moderately potent against *L. cuprina* compared to other spider toxins that affect dipterans.

Notably, Mb1a and Mb1b were inactive against *H. armigera*, suggesting they do not modulate the activity of *H. armigera* Na_V_ or Ca_V_ channels. Since a BAC library of the *H. armigera* genome has been published [51,52], we used this to deduce the sequence of the *H. armigera* Na_V_ channel and compared it to that of *B. germanica* and *P. americana* channels on which Mb1a and Mb1b are active (Figure 11). The S3–S4 loops are identical in all four voltage-sensor domains of the of *P. americana*, *B. germanica* and *H. armigera* Na_V_ channels, indicating that these regions are not responsible for the differences in activity. However, the S1–S2 loops of all four domains of the *H. armigera* Na_V_ channel contain variations in amino acid sequence compared to both cockroach species (Figure 11). Thus, Mb1a and Mb1a possibly provide additional examples of insecticidal toxins where differences in phyletic selectivity are due to taxonomic variations in the sequences of the S1–S2 loops [24,37].

## 4. Conclusions

Mb1a and Mb1b add to the expanding repertoire of spider toxins that are active against insects. Due to a high level of homology with toxins active on vertebrates, Mb1a and Mb1b may have an activity on vertebrate Na_V_ or Ca_V_ channels that would render them unsuitable candidates for bioinsecticide development. However, the closest vertebrate-active homolog is only 71% identical with 11 differences in amino acid sequence (Figure 10), and it is well known that even small differences in venom-peptide sequences can alter their channel and species selectivity. Even if Mb1a and Mb1b turn out to be vertebrate active, differences in their mechanism of action make them useful tools for studying insect Na_V_ channels. Moreover, Mb1a and Mb1b contribute to the growing body of evidence derived from animal toxins which suggests that phyletic selectivity can be achieved by targeting the voltage-sensor domains of insect Na_V_ channels.

## 5. Materials and Methods

### 5.1 Venom Collection

Venom from a single female *Monocentropus balfouri* was extracted using mild electrical stimulation [53], then the venom was lyophilized and kept frozen until reconstituted and used for the experiments.

### 5.2. Fractionation of Crude Venom

1 mg of dried *M. balfouri* venom was fractionated using a Jupiter C_18_ analytical RP-HPLC column (25 cm × 4 mm, 5 µm, Phenomenex Australia Pty. Ltd., Sydney, Australia) connected to a Shimadzu Prominence HPLC system. The flow rate was 1 mL/min. Varying gradients of solvent A (0.1% formic acid) and solvent B (90% acetonitrile/ 0.1% formic acid) were used. Isocratic elution with 5% solvent B was used for the first 5 min, followed by 5–20% solvent B over 5 min, 20–40% solvent B over 40 min, 40–80% solvent B over 5 min, then 80% solvent B for 2 min.

### 5.3. Sequencing of Active Peptide

N-terminal Edman sequencing, including the prior reduction and alkylation of Mb1a/b with TCEP/iodoethanol, was performed by the Australian Proteome Analysis Facility (APAF). The sample (30 µL) was loaded onto a precycled, biobrene-treated discs and subjected to 40 cycles of Edman N-terminal sequencing. The analysis was run using the pulsed liquid method with a modified begin cycle to include extra washes. Automated Edman degradation was carried out using an Applied Biosystems 494 Procise Protein Sequencing System. Performance of the sequencer was assessed routinely with 10 pmoL β-lactoglobulin standard.

### 5.4. Recombinant Production of Mb1a and Mb1b

Recombinant Mb1a and Mb1b were produced by expression in the periplasm of *Escherichia coli* using a previously described protocol [21]. Briefly, a synthetic gene encoding Mb1a and Mb1b was cloned into a variant of the pLic-MBP expression vector by GeneArt (Invitrogen, Regensburg, Germany). Codons were optimized for expression in *E. coli*. The modified pLic-MBP vector encodes a MalE signal sequence for periplasmic export of the fusion protein, a His_6_ tag for affinity purification, a maltose-binding protein (MBP) fusion tag to aid solubility, and a tobacco etch virus (TEV) protease recognition site directly preceding the toxin sequence. The plasmids encoding Mb1a and Mb1b were transformed via heat shock into *E. coli* strain BL21(λDE3) for production of recombinant toxin. Protein expression and purification were performed as previously described with minor modifications. In summary, a 50 mL overnight starter culture grown in Luria-Bertani broth at 37 °C with shaking (~220 rpm) was used to inoculate a 2 liter culture the following day. After the culture reached an OD_600_ of ~1.0, toxin gene expression was induced with 500 μM IPTG. Cells were grown at 30 °C overnight before centrifugation for 15 min at 10,500 *g* to obtain the cell pellet. The pellet was then reconstituted in a minimal amount of TN buffer (20 mM Tris, 250 mM NaCl, pH 8), then cells were lysed at 27 kpsi using a constant-pressure cell disruptor (TS Series Cell Disrupter, ConstantSystems Ltd., Daventry, UK).

The cell lysate was passed over a column containing Ni-NTA Superflow resin (Qiagen Pty Ltd., Chadstone, VIC, Australia) and weakly bound proteins were eluted with 15 mM imidazole in TN buffer. The MBP-toxin fusion protein was then eluted with 300 mM imidazole in TN buffer, after which the eluate was spun in a 30 kDa cut-off centrifugal filter to remove the imidazole and concentrate the protein to 5 mL. The resulting solution was diluted to 10 mL with TN buffer, then the fusion protein was cleaved overnight at room temperature using ~100 μg TEV protease in 0.6 mM and 0.4 mM reduced and oxidised glutathione, respectively [21]. 0.1% TFA was then added to precipitate the cleaved His_6_-MBP protein; the sample was then centrifuged at 17,000 *g* to pellet the precipitant. The supernatant was filtered with a 0.45 μm syringe filter (EMD Millipore, Billerica, MA, USA) before purification of recombinant Mb1a or Mb1b using semi-preparative RP-HPLC (Phenomenex Jupiter C_4_ column; 250 × 10 mm, 10 μm; flow rate 5 mL/min). Recombinant toxin was eluted using a gradient of 10–45% solvent B (0.043% TFA in 90% acetonitrile) in solvent A (0.05% TFA in water) over 30 min. Further purifications were performed using an Agilent C_18_ column (ZORBAX 300SB; 250 × 9.4 mm, 5 μm; flow rate 3 mL/min) with appropriate solvent gradients.

### 5.5. Insecticidal Assays

#### 5.5.1. Sheep Blowflies and House Flies

Mb1a and Mb1b were dissolved in insect saline (see [54] for composition) and injected into the ventro-lateral thoracic region of adult sheep blowflies (*Lucilia cuprina*) or house flies (*Musca domestica*) according to methods described previously [23]. Briefly, a maximum of 2 µL was injected per fly using a 1.0 mL Terumo Insulin syringe with a fixed 29-gauge needle fitted to an Arnold hand micro-applicator (Burkard Manufacturing Co. Ltd., Rickmansworth, UK). Flies were individually housed in 2 mL tubes and paralytic effects determined 0.5, 1, 2 and 24 h after injection. For *L. cuprina*, three replicates were performed and for each replicate six doses of Mb1a or Mb1b (*n* = 10 flies per dose) was used, along with appropriate controls (insect saline; *n* = 20 flies each). Dose-response data were fitted using the sigmoidal dose-response (variable slope) function in Prism 6. For *M. domestica*, a single replicate (*n* = 5) of one dose of Mb1a or Mb1b was injected (Mb1a: 15 nmoL/g, Mb1b: 11.7 nmoL/g). PD_50_ and LD_50_ values were calculated as previously described [55].

We defined “complete paralyzed” flies as those that could move their appendages (legs and proboscis), but were unable to fly or drag their body forward when placed onto a flat bench. At earlier times post-injection, or at lower doses, we observed “incomplete paralyzed” flies that had uncoordinated movement (e.g., due to some extremities twitching or being paralyzed) but which were nevertheless able to move their body along a flat bench even though they could not fly.

#### 5.5.2. Cotton Bollworms

Cotton bollworms (i.e., *Helicoverpa armigera* larvae) were obtained from AgBiTech Pty Ltd. (Glenvale, QLD, Australia). Toxins were injected into the lateral thoracic region and larvae observed for paralytic or lethal effects at 0.5, 1, 3, 24, 48, and 72 h after injection. Larvae were kept in standard 6-well plates and fed on artificial diet (AgBiTech, Clifford Gardens, Australia). Larval weight was measured 24, 48 and 72 h after injection.

### 5.6. Patch Clamp Electrophysiology Using P. americana Neurons

DUM neurons were isolated from unsexed adult *P. americana* as described previously [56]. Briefly, terminal abdominal ganglia were removed and placed in normal insect saline (NIS) containing (in mM): NaCl 180, KCl 3.1, 4-(2-hydroxyethyl)piperazine-1-ethanesulfonic acid (HEPES) 10, d-glucose 20. Ganglia were then incubated in 1 mg/mL collagenase (type IA) for 40 min at 29 °C, washed twice in NIS, resuspended in NIS supplemented with 4 mM MgCl_2_, 5 mM CaCl_2_, 5% foetal bovine serum and 1% penicillin/streptomycin (NIS^+^; Life Technologies, Mulgrave, VIC, Australia), then triturated through a fire-polished Pasteur pipette. The resultant cell suspension was then distributed onto 12-mm diameter glass coverslips pre-coated with 2 mg/mL concanavalin A (type IV). DUM neurons were maintained in NIS^+^ at 29 °C and 100% humidity.

Ionic currents were recorded from DUM neurons in voltage-clamp mode using the whole-cell patch-clamp technique employing version 10.2 of the pCLAMP data acquisition system (Molecular Devices, Sunnyvale, CA, USA). Data were filtered at 10 kHz with a low-pass Bessel filter with leakage and capacitive currents subtracted using P–P/4 procedures. Digital sampling rates were set between 15 kHz and 25 kHz depending on the length of the protocol. Single-use 0.8–1.5 MΩ electrodes were pulled from borosilicate glass and fire-polished prior to current recordings. Liquid junction potentials were calculated using JPCALC, and all data were compensated for these values. Cells were bathed in external solution through a continuous pressurized perfusion system at 1 mL/min, while toxin solutions were introduced via a wide-bore gravity-fed perfusion needle at ~80 μL/min (Automate Scientific, San Francisco, CA, USA). All experiments were performed at ambient temperature (20–23 °C). To record sodium currents (*I*_Na_), the external bath solution contained (in mM): NaCl 80, CsCl 5, CaCl_2_ 1.8, tetraethylammonium chloride 50, 4-aminopyridine 5, HEPES 10, NiCl_2_ 0.1, CdCl_2_ 1, adjusted to pH 7.4 with 1 M NaOH. The pipette solution contained (in mM): NaCl 34, CsF 135, MgCl_2_ 1, HEPES 10, ethylene glycol-bis(2-aminoethylether)-*N*,*N*,*N′*,*N′*-tetraacetic acid (EGTA) 5, and ATP-Na_2_ 3, adjusted to pH 7.4 with 1 M CsOH. Note that in these and other electrophysiology experiments, peptides were tested at concentrations that yielded 50–75% inhibition of currents.

Two subtypes of Ca_V_ channel currents have been observed in *P. americana* DUM neurons: high-voltage-activated (HVA) and mid/low-voltage-activated (M-LVA) Ca_V_ channel currents [23,57]. Notwithstanding differences in the kinetic and pharmacological properties of M-LVA and HVA Ca_V_ channels, there is no mechanism for recording one current in isolation from the other, as no peptide or small molecule inhibitors have been developed that block one type of current and not the other [58]. Therefore, depolarizing voltage command pulses to different levels were used to investigate the actions of Mb1a and Mb1b on M-LVA and HVA Ca_V_ channels [23,57]. Ca_V_ channel currents were evoked by 100-ms depolarising pulses from a membrane holding potential (*V*_h_) of –90 mV to potentials at 7-s intervals to −20 mV for generation of predominantly M-LVA Ca_V_ channel currents and to +20 mV to evoke mainly HVA Ca_V_ channel currents [57].

Previous studies revealed significant rundown of Ca_V_ currents when calcium was used as a charge carrier, but much less when barium was used instead [58]; thus, we replaced CaCl_2_ with BaCl_2_ in all Ca_V_ channel experiments. The external bath solution for barium current (*I*_Ba_) recordings contained (in mM): sodium acetate 140, TEA-bromide 30, BaCl_2_ 3, HEPES 10, adjusted to pH 7.4 with 1 M TEA-OH. The external solution also contained 300 nM tetrodotoxin to block Na_V_ channels. Pipette solutions contained (in mM): sodium acetate 10, CsCl 110, TEA-bromide 50, ATP-Na_2_ 2, CaCl_2_ 0.5, EGTA 10, HEPES 10, adjusted to pH 7.4 with 1 M CsOH.

To eliminate any influence of differences in osmotic pressure, all internal and external solutions were adjusted to 400 ± 5 mOsmol/L with sucrose. Experiments were rejected if leak currents exceeded 1 nA or if currents showed signs of poor space clamping. Peak current amplitude was analyzed offline using AxoGraph X v1.5.3 (Molecular Devices, Sunnyvale, CA, USA). All curve-fitting was performed using Prism 6 (GraphPad Software Inc., San Diefo, CA, USA). All data are mean ± SEM of *n* independent experiments. On-rates (τ_on_) were calculated using the following Equation (1):

(1) $$Y=Y_{0}+\left(A-Y_{0}\right)\times \left(1-\exp\left(-K\times t\right)\right)$$

where *Y_0_* is the maximal peak *I*_Na_, *A* is the minimum peak *I*_Na_, *K* is the rate constant and *t* is time. The on-rate (τ_on_) was subsequently determined from the inverse of the rate constant (*K*).

The data for voltage-dependence of channel activation, for all channel types, were fitted using the following current-voltage (*I*/*V*) curve formula Equation (2):

(2) $$I=g_{max}\left(1-\left(\frac{1}{1+\exp\left[(V-V_{1/2}\right)/s]}\right)\right)\left(V-V_{rev}\right)$$

where *I* is the amplitude of the current at a given test potential *V*, *g_max_* is the maximal conductance, *V*_1/2_ is the voltage at half-maximal activation, *s* is the slope factor and *V*_rev_ is the apparent reversal potential.

Differences in current inhibition between toxins was analysed using one-way ANOVA, with a probability of *p* < 0.05 being considered statistically significant.

### 5.7. Two–Electrode Voltage-Clamp Electrophysiology

BgNa_V_1 [59] cRNA was synthesized using T7 polymerase (mMessage mMachine kit, Life Technologies, Carlsbad, CA, USA) after linearizing the fully-sequenced DNA with *Not*I. BgNa_V_1 was expressed in *Xenopus* oocytes together with the TipE subunit [60] (1:5 molar ratio), and studied following a 1-day incubation after cRNA injection. Cells were incubated at 17 °C in ND96 consisting of (in mM) NaCl 96, KCl 2, HEPES 5, MgCl_2_ 1, CaCl_2_ 1.8, pH 7.6 with NaOH supplemented 50 μg/mL gentamycin, then studied using two-electrode voltage-clamp recording techniques (OC-725C, Warner Instruments, Hamden, CT, USA) with a 150-μL recording chamber. Data were filtered at 4 kHz and digitized at 20 kHz using pClamp software (Molecular Devices, Sunnyvale, CA, USA). Microelectrode resistances were 0.5–1 MΩ when filled with 3 M KCl. The external recording solution consisted of ND96. All experiments were performed at room temperature (~22 °C). Leak and background conductances, identified by blocking the channel with tetrodotoxin, were subtracted for all currents shown. Voltage-activation relationships were obtained by measuring steady-state currents and calculating conductance. Protocols for other measurements are described in the figure legends. After addition of toxin to the recording chamber, the equilibration between the toxin and the channel was monitored using weak depolarizations elicited at 5-s intervals. For all channels, voltage-activation relationships were recorded in the absence and presence of toxin. Off-line data analysis was performed using Clampfit 10 (Molecular Devices, Sunnyvale, CA, USA) and Origin 8.0 (Originlab, Northampton, MA, USA).
